# Minimally-Invasive Imaging of Sublingual Vessels—A New Method to Study Microvascular Changes in Mice

**DOI:** 10.3390/life15091478

**Published:** 2025-09-20

**Authors:** Ellen Dyminski Parente Ribeiro, Maryam Dastan, Ursula Bellut-Staeck, Juan Zhou, Christian Lehmann

**Affiliations:** 1 Faculty of Medicine, Federal University of Parana, Curitiba 80060-240, PR, Brazil; ellen.parente@ufpr.br; 2 Department of Anesthesia, Pain Management and Perioperative Medicine, Dalhousie University, Halifax, NS B3H 2H8, Canada; maryam.dastan@dal.ca (M.D.); juan.zhou@dal.ca (J.Z.); 3 Independent Researcher, 14089 Berlin, Germany

**Keywords:** microcirculation, sublingual vessels, vasomotion, mice, sidestream dark field imaging

## Abstract

Sepsis causes profound microcirculatory dysfunction, where heterogeneous capillary perfusion and abnormal vasomotion contribute to tissue hypoxia and organ failure. Sublingual videomicroscopy is established in large animals and humans to monitor these alterations, but this approach has not been applied in murine models. We developed a method to assess sublingual perfusion and vasomotion in mice using sidestream dark field (SDF) videomicroscopy. Male C57BL/6 mice were anesthetized, and sublingual vessels were imaged for 90 min. Perfusion and vasomotion parameters were assessed, and a Fast Fourier Transform was performed on vasomotion data to characterize the frequency components of rhythmic microvessel diameter changes. Another group of animals was administered lipopolysaccharide (LPS) intraperitoneally as a model of systemic inflammation in sepsis. LPS-treated mice exhibited a significant decline in the proportion of perfused vessels at 90 min and in the microvascular flow index. Very low-frequency vasomotion (0.005–0.15 Hz) declined in controls but was preserved during endotoxemia, suggesting an active microvascular tone response to inflammatory stress. This study establishes the first murine protocol for sublingual SDF imaging, allowing early detection of perfusion deficits and vasomotor changes in experimental sepsis. The minimal-invasive approach offers a translational platform for mechanistic and therapeutic studies in sepsis.

## 1. Introduction

Sepsis results from a dysregulated host response to infection, combining excessive inflammation with immune suppression that ultimately drives cellular dysfunction and organ failure [1]. Central to this process is the microcirculation, where injury both originates and propagates [2]. Under normal conditions, microvascular perfusion is regulated by neuroendocrine, paracrine, and mechanosensory pathways that match local oxygen delivery to metabolic demand [3].

In sepsis, these regulatory mechanisms fail as the inflammatory cascade and oxidative stress induce profound endothelial dysfunction [4]. Capillary density decreases and perfusion becomes heterogeneous, with intermittent or absent flow creating hypoxic zones and impairing tissue oxygenation even when total organ blood flow appears normal [5]. This heterogeneity compromises oxygen delivery more severely than uniform hypoperfusion and actively drives organ dysfunction rather than merely reflecting disease severity [6,7].

An important component of this regulation is vasomotion, defined as the rhythmic contraction and relaxation of small vessels that modulate blood flow and play a central role in tissue perfusion and oxygen delivery [8,9,10]. Hemodynamic oscillations resulting from vascular tone variations have been proposed as a tool to evaluate microcirculatory function [11,12,13]. Frequency analysis separates these oscillations into distinct bands linked to specific physiological mechanisms, including cardiac and respiratory activity, myogenic tone, neurogenic input, and endothelial-dependent regulation [14]. In rodent sepsis models, intravital videomicroscopy studies have demonstrated that early microcirculatory impairment is accompanied by enhanced low frequency vasomotion, reflecting dysregulated local vascular control [11,13].

Clinical studies using bedside sublingual videomicroscopy, employing orthogonal polarization spectral (OPS), sidestream dark field (SDF), and incident dark field (IDF) handheld devices, have consistently linked sepsis-induced microvascular alterations to patient outcomes [15,16,17,18]. Rapid recovery of microcirculatory function occurs in survivors of septic shock, while persistent microvascular alterations, independent of systemic hemodynamics, are strongly associated with multiple organ failure and poor outcomes [19,20]. Importantly, changes in capillary perfusion often precede abnormalities in vital signs or laboratory results, highlighting sublingual microcirculation as a sensitive early marker of systemic dysfunction [21]. Consequently, improving microcirculation through infection control, fluid resuscitation, and vasoactive support remains a mainstay of sepsis therapy [1,4].

In preclinical research, various in vivo techniques exist to assess microcirculation, each with distinct advantages and limitations depending on the experimental goals. In larger animal models such as pigs [22,23], rats [24,25], sheep [26,27,28], and rabbits [29,30,31,32], handheld videomicroscopy devices, the same technology used at the bedside in septic patients, are well established in sepsis research. These methods offer key benefits: they require no vascular catheterization or dye injection, enable rapid and straightforward setup, and provide high translational value due to their clinical applicability [33,34,35].

By contrast, murine models predominantly rely on intravital microscopy (IVM), which allows high-resolution imaging of well-vascularized tissues such as the mesentery [36] or exposed muscle [37] over short durations in anesthetized animals. IVM facilitates detailed visualization of blood flow, leukocyte–endothelial interactions, vascular permeability, and molecular dynamics using fluorescent dyes or transgenic reporter mice [33]. It also enables quantitative assessment of perfusion and flow velocity beyond surface vessels [38]. However, IVM’s invasive nature, requiring surgical exteriorization of organs or muscle, introduces mechanical stress that can disrupt physiological conditions and compromise translational relevance [39,40]. Moreover, IVM is technically demanding, costly, and less reflective of clinical monitoring practices [41].

Given these challenges, our choice of SDF imaging in this study is strategically motivated. SDF provides a non-invasive, rapid, and reproducible method that avoids surgical manipulation, thereby better preserving physiological conditions [33,34,35]. Crucially, SDF utilizes clinically established technology [33], creating a direct translational bridge that enhances the relevance of our findings to human sepsis. Additionally, SDF allows the measurement of blood flow in microvessels for perfusion assessment and the detection of diameter oscillations for vasomotion evaluation [42,43]. This combination of practicality, physiological fidelity, and clinical applicability makes SDF the optimal approach for our preclinical investigation.

Despite its translational importance, sublingual microcirculation assessment has not yet been performed in mice. While mice are prized for their low cost, genetic tractability, and wide availability [44,45], the small size of the mouse tongue and technical challenges in stabilizing it for precise imaging have hindered adoption of this approach. To overcome these challenges, we developed a minimally invasive method to evaluate systemic microcirculatory changes via the sublingual vascular bed using a lipopolysaccharide (LPS)-induced endotoxemia mouse model, one of the most widely used preclinical models of sepsis [46]. This method enabled the assessment of both perfusion and vasomotion parameters under inflammatory stress, while minimizing experimental artifacts, reducing animal burden, and enhancing the translational relevance of murine microcirculatory research.

## 2. Materials and Methods

### 2.1. Animals

All experimental procedures adhered to Canadian Council for Animal Care Guidelines and were approved by the Dalhousie University Committee on Laboratory Animals. Six male C57BL/6 mice (25–30 g; 6–8 weeks old) were housed in the Carlton Animal Care Facility at Dalhousie University’s Faculty of Medicine. Animals had ad libitum access to water and rodent chow and were maintained under standard 12-h light/dark cycles.

### 2.2. Mouse Preparation for In Vivo Imaging

General anesthesia was induced using 3.5% isoflurane in an induction chamber. Once anesthetized, the mouse was positioned on a heating pad to maintain a stable body core temperature of 37.5 °C, continuously monitored via a rectal probe. The limbs were secured to the pad using tape. Anesthesia was maintained throughout the experiment with 2% isoflurane delivered via a nose cone.

The study included two experimental groups: a control group (*n* = 3) composed of healthy animals with no intervention, and an endotoxemia group (*n* = 3) that received an intraperitoneal injection of lipopolysaccharide (LPS) (5 mg/kg; Escherichia coli, serotype O26:B6, potency 3,000,000 EU/mg, Sigma-Aldrich, Oakville, ON, Canada) following anesthesia induction. LPS was diluted in saline at a concentration of 1 mg/mL, and a dose of 5 mg/kg was administered based on each mouse’s body weight.

**Figure 1:**
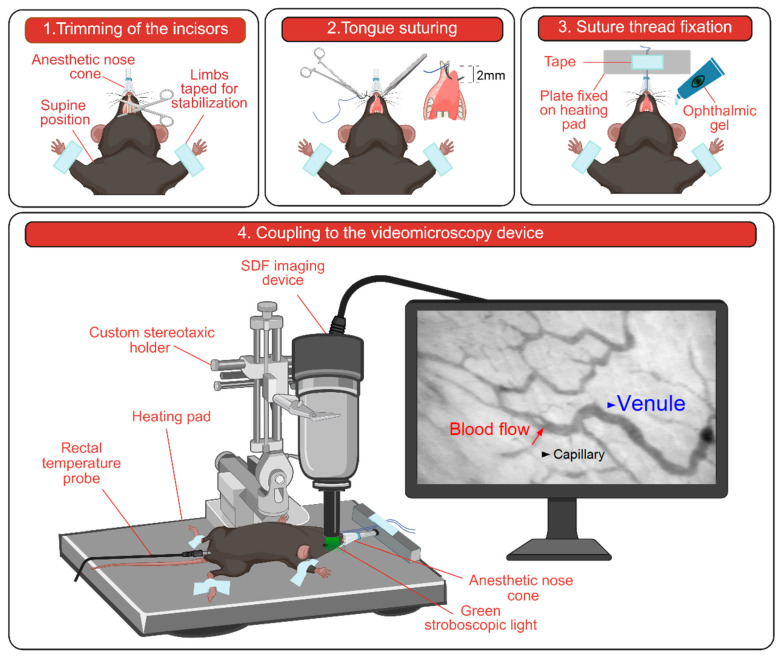
Schematic diagram of the experimental workflow. Created in BioRender. Dickson, K. (2025) https://BioRender.com/3w0wphl (accessed on 30 July 2025).

The lower surface of the tongue was prepared for microscopic imaging (Figure 1). To facilitate sublingual access and optimize camera alignment, the lower incisors were trimmed at the base using surgical scissors. The tongue was carefully extended with tweezers and secured to a holding plate using a 5-0 polyester suture placed approximately 2 mm from the apex. The suture thread was then taped to a plate positioned cranially to the mouse, orienting the lower surface of the tongue upward for imaging. Special care was taken to avoid excessive tension or pressure and to preserve physiological blood flow. To maintain tissue hydration, ophthalmic gel was applied to the tongue mucosa prior to coupling with the imaging device.

### 2.3. Setup and Image Acquisition

Live imaging of sublingual microcirculation was performed using side-stream dark-field (SDF) videomicroscopy (MicroScan, MicroVision Medical, Amsterdam, The Netherlands). This system features miniature microscope optics surrounded by light-emitting diodes (LEDs) that emit stroboscopic green light at a wavelength of 530 nm, which is selectively absorbed by hemoglobin in red blood cells while being transmitted through the surrounding tissue, thereby generating intrinsic contrast [47]. This allows for real-time visualization of the sublingual microcirculation and blood flow without the need for fluorescent dyes or exogenous contrast agents.

A modified, adjustable stereotaxic holder was used to mount the SDF probe, allowing precise positioning and mechanical stability when the probe was brought into contact with the lower surface of the tongue. During recordings, illumination and focus were continuously optimized under low ambient light to ensure high-quality visualization.

Control animals were recorded continuously for 90 min. In the LPS group, animals were recorded for 5 min to establish a baseline prior to LPS injection, followed by continuous recording for an additional 90 min post-injection.

Video output was displayed on a monitor and simultaneously routed through a FireWire signal converter (IEEE 1394) to a Windows computer, allowing direct digital recording in WinDV (http://windv.mourek.cz/, accessed on 1 July 2025, Czech Republic) of the images as AVI files on the hard drive for subsequent offline analysis.

### 2.4. Image Analysis

Microcirculatory images were analyzed for perfusion using AVA 3.0 (Automated Vascular Analysis, Academic Medical Center, University of Amsterdam, Amsterdam, The Netherlands). Following perfusion assessment, vasomotion was analyzed to capture rhythmic microvessel diameter changes, which are known to be altered in early sepsis [11,13,48]. Vasomotion analysis was performed using FIJI (version 1.54p) [49] with the VasoMetrics plugin (https://github.com/mcdowellkonnor/ResearchMacros, accessed on 6 July 2025) [50].

Vasomotion power was evaluated at seven time points (R0, 15, 30, 45, 60, 75, and 90 min), as this represented our main outcome measure. For the secondary outcome, AVA, analysis was restricted to three time points (R0, 45, and 90 min) to reduce redundancy and focus on representative phases of the response. In the LPS group, R0 corresponded to the baseline recording prior to injection.

The parameters selected to analyze perfusion in AVA 3.0 software included total vessel density (TVD), the proportion of perfused vessels (PPV), and the microvascular flow index (MFI). Blood flow in each small vessel (diameter ranging from 3.8 µm to 34.0 µm) was characterized into four categories: (0) absent, (1) intermittent (flow absent ≥ 50% of the time), (2) sluggish, and (3) continuous [51]. TVD accounted for all visible vessels, independent of flow characteristics. PPV reflected vessels exhibiting either continuous or sluggish flow. The MFI was calculated by dividing each image into four quadrants, identifying the predominant flow type within each (continuous, sluggish, intermittent, or absent), and then averaging the flow scores across all quadrants. All perfusion analyses were performed by a single investigator.

For vasomotion assessment using the VasoMetrics plugin in FIJI (version 1.54p), a midline was first drawn along a selected vessel segment. The plugin then automatically generated perpendicular cross-lines and measured the distance between vessel walls at each point. Vessel diameter was recorded for every video frame, and the resulting data were exported to an Excel spreadsheet. This produced a detailed time series of diameter fluctuations, capturing vasomotion dynamics across experimental conditions.

To analyze the frequency characteristics of vessel diameter oscillations, Fast Fourier Transform (FFT) was performed using Python (version 3.11.5, Python Software Foundation, https://www.python.org/) (Supplementary Script S1). At each time point, 5-min recordings were processed with the VasoMetrics plugin to extract time-series vessel diameter data. These data were imported into Python (version 3.11.5) and transformed into the frequency domain via FFT. For each frequency band, the mean spectral magnitude of oscillations was calculated from the modulus of the FFT coefficients. In line with previous vasomotion literature [12,14], these values will hereafter be referred to as spectral power, although they represent spectral magnitude rather than true power (squared magnitude). This approach enabled visualization of vasomotion across frequency bands associated with distinct physiological mechanisms in mice: very low frequency (VLF; 0.005–0.15 Hz), linked to endothelial, sympathetic, and myogenic activity; low frequency (LF; 0.15–2 Hz), related to respiratory influences; and high frequency (HF; 2–8 Hz), corresponding to cardiac activity [52].

### 2.5. Statistics

Normality of the data was assessed using the Shapiro-Wilk test and confirmed for TVD and vasomotion power. A two-way ANOVA was performed to analyze the data. Also, at the 90-min mark, comparisons of vasomotion power and TVD between the control and LPS-injected groups were performed using one-tailed unpaired *t*-tests with Welch’s correction. As MFI and PPV did not follow a normal distribution, group comparisons at each time point were conducted using the non-parametric Mann-Whitney test. Statistical significance was set at *p* ≤ 0.05. For each variable, graphs display the mean and standard deviation. All statistical analyses and graph generation were performed using GraphPad Prism version 10.3.1 (GraphPad Software Inc., Boston, MA, USA).

## 3. Results

### 3.1. Microcirculatory Perfusion

**Figure 2:**
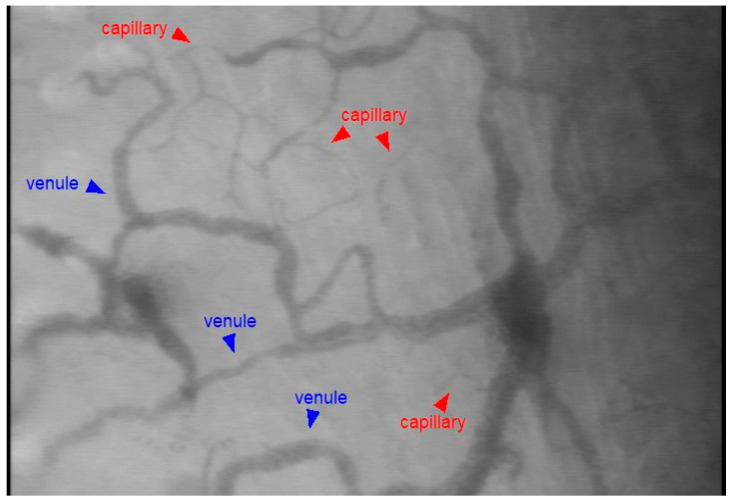
Representative image of sublingual microcirculation in mice obtained by SDF imaging.

**Figure 3:**
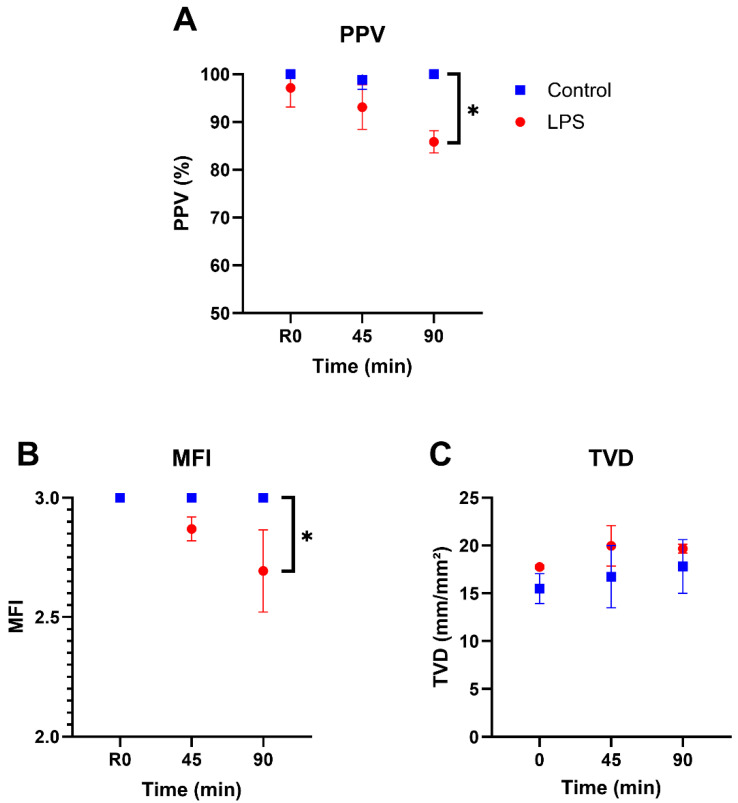
Perfusion parameters. (**A**) PPV over time in control and LPS-injected groups. (**B**) MFI over time in control and LPS-injected groups. (**C**) TVD over time in control and LPS-injected groups. LPS—lipopolysaccharide; MFI—microvascular flow index; PPV—proportion of perfused vessels; TVD—total vessel density. * Indicates *p* ≤ 0.05.

Figure 2 shows a representative still image of the sublingual microcirculation obtained with our method. Throughout the 90-min observation period, PPV in the control group remained close to 100%, whereas LPS-injected animals exhibited a decline beginning at 45 min, reaching a mean of 85.8% at 90 min (Figure 3A). At 90 min post-injection, the LPS group showed a significantly lower PPV compared to controls (*p* = 0.0045), while the difference at 45 min was not statistically significant (*p* = 0.0772). Supplementary Videos S1 and S2 show sublingual microcirculation recordings at baseline and 90 min after LPS injection, respectively.

The MFI in control animals remained constant at the maximum score of 3.0 throughout the 90-min observation period, indicating continuous blood flow in all four quadrants (Figure 3B). In contrast, LPS-injected animals exhibited a significant decline in MFI, reaching a mean of 2.69 ± 0.17 at 90 min (*p* = 0.0500).

TVD values remained comparable between groups throughout the 90-min observation period, with no significant differences at baseline (*p* = 0.0626), 45 min (*p* = 0.1171), or 90 min (*p* = 0.1839) (Figure 3C).

### 3.2. Vasomotion

**Figure 4:**
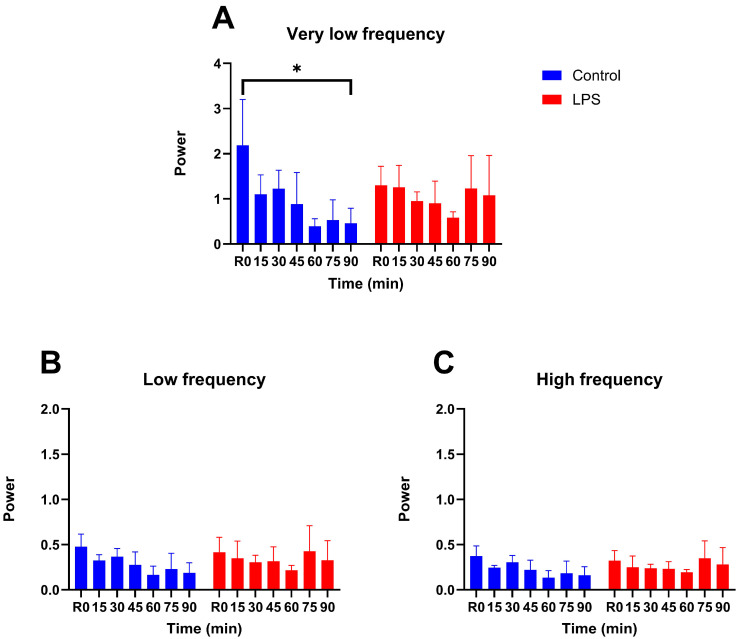
Average spectral power of vasomotion per frequency band. (**A**) Very low frequency: 0.005–0.15 Hz. (**B**) Low frequency: 0.15–2 Hz. (**C**) High frequency: 2–8 Hz. LPS—lipopolysaccharide. * Indicates *p* ≤ 0.05.

In the control group, vasomotion power in the very low frequency band progressively declined, reaching less than half of its initial value by the end of the 90-min observation period and showing a significant difference from baseline (one-tailed *t*-test, *p* = 0.0426; Figure 4A). In contrast, in the LPS group, vasomotion power in this band remained consistently close to baseline throughout the experiment, with no significant difference between baseline and 90 min. For the other frequency bands, vasomotion power at 90 min was comparable between groups in both the low-frequency (one-tailed *t*-test, *p* = 0.1974; Figure 4B) and high-frequency (one-tailed *t*-test, *p* = 0.1956; Figure 4C) bands. Two-way repeated measures ANOVA revealed no significant effects of time, group, or their interaction in any frequency band.

## 4. Discussion

In this study, we evaluated the utility of a minimally invasive SDF videomicroscopy technique targeting sublingual vessels to detect early microcirculatory alterations in murine endotoxemia as an experimental model of sepsis. Our results offer valuable insights into how perfusion and vasomotion are affected during systemic inflammation.

Significant perfusion changes were observed in the LPS group compared to controls by 90 min post-injection, with the proportion of PPV beginning to decline at 45 min and reaching a mean of 85.8%, consistent with findings from both clinical [20,53] and experimental studies [30,54,55]. A decrease in MFI was also observed in the LPS group, in contrast to the preserved flow seen in controls. The absence of significant changes in TVD over the 90-min period is consistent with studies showing that reductions in vascular density often require longer durations to become evident, typically around 4 h after sepsis induction [55].

Microcirculatory perfusion impairment is an early hallmark of sepsis and has been closely linked to organ failure [21]. This dysfunction frequently precedes detectable changes in systemic hemodynamics and is characterized by the coexistence of well-perfused and poorly perfused capillaries in close proximity, a phenomenon known as perfusion heterogeneity [56]. Mechanistically, microcirculatory dysfunction in the early stages of sepsis arises from a combination of nitric oxide (NO) dysregulation, impaired intercellular signaling, and disrupted interactions among circulating blood components [21,57]. The endotoxemia model used in this study effectively reproduces key early pathophysiological events underlying microcirculatory disturbances driven by an exaggerated inflammatory response [58,59,60]. Upon binding to Toll-like receptor 4 (TLR4), endotoxin activates signaling pathways leading to the release of proinflammatory mediators such as NF-κB, IL-2, and TNF-α, initiating a cascade that destabilizes vascular homeostasis [58,61].

One of the earliest consequences is dysregulated NO production [61]. Excessive NO synthesis via inducible nitric oxide synthase (iNOS) results in loss of vascular tone, with heterogeneous vasodilation promoting inappropriate shunting of blood away from hypoxic tissues [62,63,64]. Concurrently, endothelial cells lose their ability to regulate arteriolar smooth muscle tone, while the smooth muscle cells themselves become less responsive to adrenergic stimulation and lose contractile control [65,66,67]. Red blood cells also fail to perform their adaptive role in oxygen sensing and vasodilation, becoming less deformable and more adhesive, further compromising capillary perfusion [21,68,69]. Collectively, these mechanisms account for the early microcirculatory alterations induced by LPS [66,70], aligning with the timeframe investigated in this study and explaining the observed reductions in PPV and MFI.

In later stages of sepsis, sustained inflammation drives leukocyte and platelet activation, microthrombosis, and capillary plugging [13,57]. However, these mechanisms do not fully explain early-phase microcirculatory impairment, which appears primarily mediated by inflammation-induced vascular dysfunction rather than mechanical obstruction [13,57]. Although earlier studies implicated capillary plugging in the initial response to sepsis, evidence has repeatedly failed to support this hypothesis [11,13]. Microvascular flow disturbances can precede significant capillary blockage, highlighting the central role of endothelial and vascular smooth muscle dysfunction in early tissue perfusion deficits [11,13]. Furthermore, impaired signaling from capillaries to upstream arterioles may limit oxygen delivery to metabolically active tissues, exacerbating early microcirculatory dysfunction [13,57]. While the LPS model lacks features of clinical sepsis such as ongoing infection and host–pathogen interactions, it remains a valuable and reproducible tool for investigating early immune and microvascular alterations characteristic of sepsis.

In this context, we examined dynamic changes in vasomotion, a key feature of microvascular regulation that may reflect early functional disturbances in the absence of structural damage. Two observations regarding vasomotion were particularly noteworthy. First, in the control group, the average spectral power in the very low frequency band progressively declined over the 90-min period, falling to less than half of its initial value and showing a significant decrease from baseline. We hypothesize that the progressive decline in very low frequency power in the control group may reflect reduced endothelial or neurovascular tone over time. This could indicate recovery from initial stress due to anesthesia [70] and tongue manipulation, which may transiently enhance vasomotion through endothelial dysfunction and vasoactive mediators [71,72].

Second, in the LPS group, spectral power in the very low frequency band remained consistently close to baseline throughout the experiment, indicating relatively elevated vasomotion compared to the control group at both 45 and 90 min. Given the established association of this frequency range with endothelial, sympathetic, and myogenic activity [72], such preservation suggests that LPS sustains or enhances local microvascular regulatory mechanisms in response to systemic inflammation. During sepsis, initial peripheral vasoconstriction reduces blood flow, potentially leading to hypoxia in arteriolar vascular smooth muscle cells and impairing their ability to maintain tone [21]. As iNOS expression increases, elevated NO levels promote vasodilation, restoring red blood cell flow and oxygenation, and enabling the reinitiation of constriction [21]. This alternating pattern of vasoconstriction and vasodilation gives rise to pseudo-periodic vasomotion, a phenomenon thought to enhance local oxygen delivery and optimize tissue perfusion under critically hypoperfused conditions [9,73]. Although this compensatory mechanism may help improve microvascular flow, particularly near the arteriolar network, it comes at a cost. By lowering systemic vascular resistance, it can contribute to the persistent hypotension that characterizes septic shock, often refractory to fluid resuscitation [9,74,75].

Notably, the low-frequency (0.15–2 Hz) and high-frequency (2–8 Hz) bands, linked to respiratory and cardiac influences, respectively, remained stable across groups, indicating that systemic cardiorespiratory modulation of vasomotion was not significantly impacted by LPS in the 90 min of the experiment. These findings align with previous reports that local microvascular activity, particularly endothelial-driven oscillations, may respond more sensitively to pathophysiological stimuli than systemic vital signs alone [14,76].

An important consideration in the interpretation of our findings is the effect of anesthesia on microcirculation. Anesthesia is well known to influence both perfusion and vasomotor activity, typically leading to a reduction in the proportion of perfused vessels and a suppression of spontaneous vasomotion, although the magnitude of these effects depends on the specific anesthetic agent and experimental context [77,78,79]. Isoflurane alters microcirculatory parameters across species, including mice [80,81]. In a mouse model using dorsal skinfold chambers, researchers compared propofol with isoflurane and found that isoflurane significantly reduced microvascular flow at 120 min post-sedation (mean MFI: 1.6 ± 0.9 under isoflurane vs. 2.8 ± 0.3 under propofol; *p* < 0.001), indicating that isoflurane may impair microvascular perfusion more substantially than propofol [81]. Additionally, laser Doppler perfusion imaging studies in mice show that 1.5% isoflurane provides stable peripheral perfusion over time, with minimal hemodynamic disturbance, making it acceptable for use as a reference baseline in microcirculatory investigations [82]. In the cerebral microvasculature, high-resolution optical coherence tomography imaging disclosed a strong vasodilatory response to isoflurane: arterial diameter increased by 12–55%, vessel blood flow surged by approximately 96%, and tissue perfusion rose by about 85% [83].

Isoflurane’s impact on vasomotion is both vessel size and endothelium-dependent. In rabbit coronary arteries, it caused vasodilation in large conductance vessels (mediated by nitric oxide and cyclooxygenase products) but vasoconstriction in smaller resistance microvessels, with both effects requiring an intact endothelium [84]. Similarly, in endotoxemic rodents, isoflurane nearly abolished arteriolar vasodilation, indicating impaired vasoregulatory capacity under septic conditions [85]. In the brain, isoflurane increased microvascular flow in a dose-dependent fashion, more prominently than sevoflurane, while attenuating autoregulation at higher concentrations [86,87]. To date, however, there are no studies specifically investigating isoflurane’s direct effects on vasomotion in mice. Nevertheless, given the conserved mechanisms of endothelial-dependent vasoregulation across species, it is reasonable to assume that similar modulatory effects are present in the murine microcirculation [81].

Crucially, in our study, both the control and LPS-treated mice were exposed to identical isoflurane anesthesia conditions. Despite this shared baseline, we still observed significant differences in sublingual microvascular perfusion and vasomotion between groups, underscoring the conclusion that the observed alterations primarily reflect the effects of LPS-induced inflammation rather than anesthesia alone. Isoflurane is widely validated in murine microcirculation studies for maintaining stable hemodynamics and allowing reliable comparison between experimental groups.

To the best of our knowledge, this is the first study to assess microcirculation and vasomotion in the sublingual vessels of mice, introducing a novel and minimally invasive approach to evaluate systemic microcirculatory parameters in an easily accessible vascular bed. The use of the same handheld videomicroscopy technique applied in larger animal models and humans enhances the translational potential of preclinical research by enabling direct cross-species comparisons and improving clinical relevance.

This study has several limitations. First, although animal models are essential for understanding the pathophysiology of sepsis, they do not fully replicate human physiology. Specifically, the use of LPS induces sepsis-like changes much more rapidly than occurs in the natural progression of human disease. Moreover, our model did not include any therapeutic interventions, such as antibiotics or fluid resuscitation, which limits its translational relevance to the complex clinical management of sepsis in hospitalized patients. Another point is that control animals did not receive a saline injection. However, the small extra amount of volume in LPS animals due to the saline vehicle might have, if at all, improved their condition. Therefore, the observed significant group differences at the 90-min mark are even more meaningful. Technical limitations related to intravital imaging must also be acknowledged. Factors such as a restricted field of view, motion artifacts, pressure sensitivity, and limited frame rate may influence the accuracy and reproducibility of microcirculatory measurements, even when bias-reducing strategies are employed. Finally, while the specific SDF imaging device used in this study is no longer commercially available, comparable tools such as the incident dark field (IDF) video microscopy (CytoCam, Braedius Medical, Huizen, The Netherlands) continue to offer viable alternatives for microcirculatory assessment [88].

Although this study utilized an SDF camera to visualize real-time microvascular flow, the setup can be adapted for other imaging modalities, such as optical and two-photon fluorescence microscopy. These techniques, previously applied to the dorsal tongue to study taste bud physiology [89,90] and tumor invasion [91], could allow for simultaneous visualization of vascular and immune responses under various stressors. The procedure could also be refined to enable brief survival imaging by avoiding tooth trimming, gently positioning the tongue with tweezers, and replacing sutures with suction-based fixation.

Finally, future work should integrate additional microhemodynamic parameters, such as tissue oxygenation, hemoglobin content, blood pressure, and lactate levels, to better characterize their relationship with sublingual perfusion and vasomotion during early sepsis. Moreover, future studies should incorporate immune parameters, including cytokine measurements, to correlate systemic inflammation with microcirculatory changes. Larger cohorts and inclusion of female animals would improve statistical power and allow assessment of potential sex-specific differences. Validation of these techniques against established methods will also be essential to confirm their accuracy and define meaningful metrics for assessing sublingual microvascular function. The translational potential could also be strengthened by testing whether MFI, PPV, and PVD are as reliable predictors of outcomes (e.g., mortality, organ failure) in experimental models using different LPS doses, as has been consistently shown in septic patients.

## 5. Conclusions

This study demonstrated the feasibility of using a minimally invasive SDF videomicroscopy technique to assess sublingual microcirculation and detect early microcirculatory impairment during LPS-induced endotoxemia in mice. It also revealed that sublingual vasomotion dynamics may exhibit increased power following immune challenge with LPS, potentially reflecting early compensatory mechanisms of microvascular regulation.

## Data Availability

The raw data supporting the conclusions of this article will be made available by the authors on request.

## Supplementary Materials

The following supporting information can be downloaded at: https://www.mdpi.com/article/10.3390/life15091478/s1, Script S1: Fast Fourier Transform analysis script and related files (analysis script, configuration, example data, README); Video S1: Baseline video available in MP4 and AVI formats; Video S2: After LPS injection video available in MP4 and AVI formats.
