# Correction: Dyminski Parente Ribeiro et al. Minimally-Invasive Imaging of Sublingual Vessels—A New Method to Study Microvascular Changes in Mice. *Life* 2025, *15*, 1478

**DOI:** 10.3390/life16020260

**Published:** 2026-02-03

**Authors:** Ellen Dyminski Parente Ribeiro, Maryam Dastan, Ursula Bellut-Staeck, Juan Zhou, Christian Lehmann

**Affiliations:** 1 Faculty of Medicine, Federal University of Parana, Curitiba 80060-240, PR, Brazil; ellen.parente@ufpr.br; 2 Department of Anesthesia, Pain Management and Perioperative Medicine, Dalhousie University, Halifax, NS B3H 2H8, Canada; maryam.dastan@dal.ca (M.D.); juan.zhou@dal.ca (J.Z.); 3 Independent Researcher, 14089 Berlin, Germany; drmed.u.bellut@t-online.de

In the published article [1], there was an error regarding the affiliation of Ursula Bellut-Staeck. She should be classified as an Independent Researcher instead of as affiliated with Dalhousie University (affiliation 2). The authors state that the scientific conclusions are unaffected. This correction was approved by the Academic Editor. The original publication has also been updated.
